# Effect of injecting adipose stem cells combined with platelet-rich fibrin releasate at Shenshu acupoint (BL23) on acute kidney injury in rabbits

**DOI:** 10.3389/fphar.2025.1409056

**Published:** 2025-03-12

**Authors:** Hsin-Ni Chuang, Wen Pei, Tzong-Fu Kuo, Yu-Hao Liu, Chia-Yih Wang, Yen-Wei Chang, Chi‐Hsuan Chuang, Chang-Huan Yang, Ming-Hsi Chuang

**Affiliations:** ^1^ Ph.D. Program of Management, Chung Hua University, Hsinchu, Taiwan; ^2^ College of Management, Chung Hua University, Hsinchu, Taiwan; ^3^ School of Veterinary Medicine, National Taiwan University, Taipei, Taiwan; ^4^ Department of Medical Research, Taipei Veterans General Hospital, Taipei, Taiwan; ^5^ Department of Cell Biology and Anatomy, College of Medicine, National Cheng Kung University, Tainan, Taiwan; ^6^ Department of Physical Education, Asia University, Taichung, Taiwan; ^7^ Genomics Research Center, Academia Sinica, Taipei, Taiwan; ^8^ Gwo Xi Stem Cell Applied Technology Co., Ltd., Hsinchu, Taiwan; ^9^ Institute of Biopharmaceutical Science, National Yang Ming Chiao Tung University, Taipei, Taiwan; ^10^ School of Public Health, National Defense Medical Center, Taipei City, Taiwan

**Keywords:** adipose derived mesenchymal cells, platelet rich fibrin, acupuncture, acute kideny injury, regenerative therapy

## Abstract

**Introduction:**

Acute kidney injury (AKI) is a major and unmet medical need, characterized by a sudden onset of kidney dysfunction that often occurs within 7 days. Adipose-derived stem cells (ADSCs) are known for their regenerative, differentiative, and repair abilities, making them a promising therapeutic option for kidney injury. Platelet-rich fibrin releasate (PRFr), derived from platelet-rich fibrin after static incubation, contains numerous growth factors that may promote the differentiation and proliferation of stem cells. Additionally, acupoints such as Shenshu (BL23) have been used in clinical practice and experimental settings, particularly in renal failure treatments.

**Methods:**

This study aimed to evaluate the synergistic effects of ADSCs and PRFr, administered separately or in combination, at the Shenshu acupoint (BL23) in New Zealand white rabbits with acute kidney injury. The treatment groups were injected with ADSCs, PRFr, or a combination of both. Serum creatinine (CRE) and blood urea nitrogen (BUN) levels were measured to assess kidney function. Additionally, histological examination of kidney tissue was performed to observe morphological changes and tissue repair.

**Results:**

The PRFr + ADSCs treatment group exhibited a significant reduction in CRE and BUN levels during the second week following transplantation. After 7 weeks of treatment, the PRFr + ADSCs group showed the most favorable kidney repair outcomes, with intact glomeruli, no edema or vacuole-like changes in the renal tubular epithelial cells, and no significant infiltration of inflammatory cells in the surrounding tissues.

**Discussion:**

The administration of PRFr, ADSCs, and their combination at the Shenshu acupoint (BL23) demonstrated a potential therapeutic effect in repairing damaged renal cells, improving kidney function, and reducing serum CRE and BUN levels. These findings suggest that injection of PRFr, ADSCs, and their combination at the Shenshu acupoint (BL23) can effectively repair damaged renal cells and improve kidney function in AKI. The observed synergistic effect indicates that this approach holds potential as a novel therapeutic strategy for kidney injury. Further research is needed to optimize treatment protocols and elucidate the underlying mechanisms.

## Highlights

Injection of PRFr + ADSCs at Shenshu acupoint (BL 23) can decrease serum CRE and BUN levels and repair damaged renal cells.

## 1 Introduction

Kidney diseases commonly observed in clinical practice include acute and chronic glomerulonephritis, renal lithiasis, renal failure, uremia, and nephrotic syndrome. Nephrotic syndrome is mostly caused by immune diseases such as primary glomerulonephritis, lupus erythematosus, and diabetic nephropathy. When the kidneys fail to perform their normal functions, waste products (toxins) and water accumulate in the body, leading to renal tubule dilation, glomerulosclerosis, proteinuria, and even kidney interstitial fibrosis, ultimately resulting in renal failure. Acute kidney injury (AKI) is caused by a decrease in renal blood flow (renal ischemia), exposure to substances harmful to the kidney (nephrotoxicity), kidney inflammation, or obstruction of the urinary tract blocking urine flow, with kidney dysfunction often occurring suddenly within 7 days (Levey and Coresh, 2012). Chronic kidney disease (CKD) refers to impaired kidney function for more than 3 months with the damage to kidney function occurring slowly and progressively. These patients require dietary control and drug treatment. In severe cases, alternative therapies are needed to replace the kidney functions, such as kidney transplantation and hemodialysis (Levey and Coresh, 2012).

In 2015, Chief Shang-Jyh Hwang, chairman of the Taiwan Society of Nephrology and a member of Kaohsiung Medical University Chung-Ho Memorial Hospital, published an analysis of the health insurance database (Lin et al., 2015). The article pointed out that refined and concentrated traditional Chinese medicine (TCM) that was scientifically prescribed is useful for treating kidney disease, with some TCM showing protective effects (Lin et al., 2015). In Taiwan, TCM has long been used to treat nephropathy. Chen-Chang Yang analyzed the health insurance database and found that patients with kidney disease who took TCM prescribed by TCM physicians had a 40% lower mortality rate than those not prescribed TCM (Hsieh et al., 2014). The study showed that joint treatment of CKD using western medicine and TCM improved patient symptoms and reduced mortality rate. Modern medicine has acquired detailed information on the pathological mechanisms of kidney disease and disease progression, and the general public has come to understand that kidney disease can be improved using TCM (Lin et al., 2015; Hsieh et al., 2014). Acupoint therapy is a treatment method in TCM of selecting acupoints along the meridian and includes acupuncture (Nie et al., 2015; Zuo et al., 2014), electroacupuncture (Zhang et al., 2014), medicinal moxibustion (Cao et al., 2010), and acupoint injection (Huang et al., 2019). This type of adjuvant therapy can improve renal function and the clinical symptoms of CKD (Huang et al., 2018; Zhong et al., 2013).

Regenerative medicine involves using healthy cells to repair or replace damaged or necrotic cells, as well as tissues or organs damaged by diseases or trauma.Stem cells have the potential to proliferate and differentiate into one or several types of cells. High concentrations of platelet-rich fibrin (PRF) contain large amounts of growth factors and cytokines that can stimulate cell regeneration and tissue repair. In recent years, the potential of stem cell technology has been widely examined (Xu et al., 2017; Savio-Silva et al., 1992). Our team developed a preparation combining PRF releasate (PRFr) and stem cells based on the potential of adipose-derived stem cells (ADSCs) to proliferate and differentiate into one or multiple cell types; we also evaluated the growth factors and cytokines present in PRFr such as platelet-derived growth factor, transforming growth factor, vascular endothelial growth factor (VEGF), epithelial growth factor, insulin-like growth factor, fibroblast growth factor, and nerve growth factor. We also developed procedures for introducing ADSCs and PRFr by injection to treat AKI caused by ischemia reperfusion (IR). Acute kidney injury in rats is induced by modeling the changes in renal function caused by hemodynamic changes (Singh et al., 2012). Cell regeneration and tissue repair are stimulated to slow the deterioration of acute kidney injury (AKI) and improve the resulting abnormal renal function. The serum creatinine (CRE) and blood urea nitrogen (BUN) concentrations, along with histological evidence, were used to evaluate treatment efficacy.

Recent studies have shown that proteins secreted by stem cells enhance tissue regeneration (Huuskes et al., 2015; Choi et al., 2023; Zhang et al., 2023). Numerous growth factors degrade rapidly after being injected into the body in liquid form. Effective delivery and controlled release of secreted factors at the injury site would improve the efficacy of tissue regeneration. Yim et al. developed a gel delivery system using conditioned medium (CM) secreted by human placental stem cells to deliver nutritional factors and evaluated the effects of these factors on kidney regeneration (Yim et al., 2019). The authors used platelet-rich plasma (PRP) gel as the delivery vehicle for CM and CM delivery to achieve controlled release of nutritional factors *in vitro* and *in vivo*, resulting in significantly enhanced cell proliferation and survival *in vitro*. In a rat model of AKI, functional and structural analysis showed that using the PRP gel system to deliver CM to the injured kidney minimized kidney tissue damage, and nutritional factors derived from human placental stem cells repaired the damaged kidney tissue (Yim et al., 2019).

In recent years, acupoints such as Sanyinjiao (SP6) (Zhang et al., 2014), Taixi (KI3) (Zhang et al., 2014), Shenshu (BL23) (Zhang et al., 2014; Cao et al., 2010; Huang et al., 2019) have been used in electroacupuncture and acupoint injection. Other acupoints, including Zusanli (ST36) (Cao et al., 2010), Pishu (BL20) (Huang et al., 2019), and Mingmen (GV4) (Huang et al., 2019), have also been used in clinical practice and experimental animals with CKD. We evaluated the synergistic effect of stem cells and growth factors by selecting the Shenshu acupoint (BL23). ADSCs ± PRFr were injected into New Zealand white rabbits with AKI induced by ischemia-reperfusion (IR) to assess the potential of this approach in treating functional failure caused by kidney disease.

## 2 Materials and methods

This project used male New Zealand white rabbits to establish an animal model of renal failure through IR injury. After anesthesia, a surgical incision was made on the flank to expose the kidney, and the renal artery was clamped for 45 min with a hemostatic forceps wrapped with a thin plastic catheter at the front, after which the wound was sutured. When selecting acupoints, a mixture of ADSCs and PRFr were injected multiple times at the rabbit’s Shenshu acupoint BL23, located at the second lumbar spinous process adjacent to open 1.5 cm (Harrison and Churgin, 2022; Chen et al., 2019).

### 2.1 Experimental animals

The Institutional Animal Care and Use Committee of National Taiwan University approved the animal experiment protocol and surgical procedures (NTU105-EL-00065). Twenty-seven New Zealand white rabbits (10 weeks old, weighing approximately 2 kg) were used. All experiments were performed in accordance with the guidelines for the care and use of laboratory animals specified by the International Council for Laboratory Animal Science.

### 2.2 ADSCs separation, culture, and identification

ADSCs were prepared as the previously report (Hsu et al., 2018). Briefly, ADSCs were obtained from the bilateral inguinal fat of New Zealand white rabbits, washed with phosphate-buffered saline (PBS), and cut into pieces. The extracellular matrix was digested (37°C, 30 min) with 0.2% collagenase type 1 (C-0130, Sigma-Aldrich Co., United States), filtered through a 100-μm membrane, and centrifuged to obtain the stromal vascular fractions. The precipitate was suspended in Dulbecco’s modified Eagle’s medium (119,995–092, Gibco., USA) containing 10% fetal bovine serum (26,140–079, Gibco., United States) and 1% penicillin-streptavidin (15,140–122, Gibco., United States), and the cells (5 × 10^4^ cells/cm^2^) were cultured at 37°C and 5% CO_2_, with the medium changed every 2 days. To collect the ADSCs, CD-90 was used for cell selection. Second-generation Cells were incubated with a mouse anti-rabbit CD90-fluorescein isothiocyanate monoclonal antibody (MA1-80648, Thermo., USA) on ice for 15 min. After washing and centrifugation, the second-passage cells were sorted using a flow cytometer (FACS Aria, Becton Dickinson., United States). Second-passage cells (Day 4) were incubated with mouse anti-rabbit CD90-FITC (Fluorescein isothiocyanate, FITC) monoclonal antibody (MA1-80648, CD90/Thy-1 monoclonal antibody, FITC conjugate, Thermo) for 15 min on ice. After washing and centrifugation, CD-90 positive cells were sorted, cultured, and passaged, with a total of 5 generations of cells from initial used for the experiments. When the cell culture volume reached 60%–70%, the cells were trypsinized, collected and incubated with the secondary antibody at 4°C for 30 min, and then washed twice with PBS containing 2% fetal bovine serum. All cell sorting and analysis were performed on the FACS Aria. The cell surface markers CD31 (ab9498, Abcam., British), CD44 (ab119335, Abcam., British), CD45 (MCA808GA, Bio-Rad., United States), and CD90 (554,895, BD Biosciences., United States) were identified. ADSCs were positive for CD44 and CD90 and negative for CD31 and CD45.

### 2.3 PRFr preparation

PRFr were prepared as the previously report (Hsu et al., 2018). Briefly, before blood collection, the rabbit was administered an intramuscular injection of a mixture (1:1) of zolazepam (12.5 mg/kg, Zoletil^®^ 50, Virbac Laboratories, France) and xylazine (10 mg/kg, Balanzine, Health-Tech Pharmaceutical Co., Taiwan) at a dose of 0.25 mL/kg for general anesthesia. The rabbit hair around the neck was shaved and thoroughly disinfected. Blood (6 mL per tube) was drawn from the right jugular vein into a blood collection tube (367,988, BD Vacutainer®SST™., United States) with gel and clot activator. The vacuum blood collection tube was slowly rotated for 30 s, and then centrifuged using a laboratory centrifuge at 3,000 rpm (1,066 × *g*) for 10 min to separate the yellow gel of the PRF from the gel and clot. After centrifugation, the fibrin clot (PRF) was removed from the upper transparent yellow serum layer and transferred into a 15-mL sterile centrifuge tube using tweezers, where the jelly-like PRF was incubated for at least 5 h as our previous study (Burnouf et al., 2012). After centrifuging the PRF at 5,000 rpm (2,962 × *g*) for 10 min, the supernatant, i.e., PRFr, was collected into a sterile vial and stored at −20°C until use. All preparation and delivery steps were performed under standard disinfection procedures. Generally, 1 mL of whole blood produced approximately 0.2 mL of PRFr.

### 2.4 Fluorescent labeling of stem cells

Fluorescent dyes can be used to label cells and track cell movement (Xing et al., 2014). To track adipose stem cells after acupoint injection, 2 × 10^6^ cells/mL of ADSCs were tagged with 10 μM of fluorescent cell tracking probe CellTracker™ Green CMFDA (C7025, Thermo Fisher Scientific Inc., United States) at 37°C for 30 min. After further centrifugation, the cells were resuspended in PBS and kept on ice until infusion.

### 2.5 Tracking fluorescent-labeled ADSCs after acupoint injection

The hehlthy rabbit was administered an intramuscular injection of a mixture (1:1) of zolazepam (12.5 mg/kg) and xylazine (10 mg/kg) at a dose of 0.25 mL/kg for general anesthesia. The rabbit hair at the Shenshu acupoint (BL23), located at the second lumbar spinous process adjacent to open 1.5 cm (Harrison and Churgin, 2022; Chen et al., 2019), was shaved and the area was thoroughly disinfected. The laboratory personnel held the rabbit in place, and then 0.5 mL of ADSCs (2 × 10^6 cells/mL) labeled with CMFDA was injected (Supplementary Figure 1). At 24 h after acupoint injection at BL23, the rabbits were sacrificed by overdose intravenous injection of citosol (Shinlin Sinseng Pharmaceutical Co., Ltd., Taiwan) and the kidney tissue was obtained to prepare frozen sections. DAPI (D9542, Sigma-Aldrich., United States) staining of the section slices was performed to detect the nucleus. A fluorescent microscope (Axiovert 40 CFL Microscope, Carl Zeiss, Germany) was used to observe whether the fluorescent cells entered the kidney tissue from the acupuncture site.

### 2.6 IR-AKI rabbit models

The commonly used clinical diagnostic criteria for AKI were formulated by Kidney Disease: Improving Global Outcomes (KDIGO) (Ketteler, 2024), which state that AKI should be diagnosed if any of the following conditions are met: (1) increase in serum creatinine of 0.3 mg/dL after 48 h; (2) increase in serum creatinine of 1.5-fold 7 days before disease onset; or (3) urine volume <0.5 mL/kg/h for more than 6 h. No previous study has proposed diagnostic criteria for AKI in rabbits, and other diagnostic criteria could not be directly applied because of species differences. The KDIGO criteria were adopted to determine establishment of the AKI models: (1) increase in serum creatinine of 0.3 mg/dL after 48 h and (2) increase in serum creatinine of 7-foldat 1 week after establishing the AKI model. The animal model of acute kidney injury in rabbits was established as in our previously published paper (Xing et al., 2014). Briefly, New Zealand white rabbits (10 weeks old, approximately 2 kg) were placed under general anesthesia as described in section 2.3. After the abdomen was shaved and disinfected, an incision was made along the middle white line of the rabbit’s abdomen through the skin and peritoneum to expose the kidneys. After identifying the renal artery, hemostatic forceps with a thin plastic catheter at the front was used to clamp this artery for 45 min before it was released Supplementary Figure 2), followed by suture of the wound using 3–0 suture (Vicryl 3.0, Ethicon, Inc., Somerville, NJ, United States). The skin gap on the abdomen was closed with interrupted sutures. Iodine was applied, and the wound was dressed.

### 2.7 Acupoint injection of ADSCs and treatment of AKI using PRFr

Twenty-four New Zealand white rabbits with AKI were randomly divided into 4 groups: control group (PBS, 0.5 mL), treatment group with PRFr (0.5 mL), ADSCs (2 × 10^6^ cells/PBS, 0.5 mL), and PRFr + ADSCs (2 × 10^6^ cells/PRFr, 0.5 mL). The injection was started at 1 week after the operation and continued for four consecutive weeks. The rabbits received BL23 acupoint injection once per week (at one inch on the left and right side of the second lumbar vertebrae), and their physiological conditions were observed until 7 weeks after the operation.

### 2.8 Biochemical analysis of serum CRE and BUN

Before the weekly acupoint injections, blood was collected from the ear artery of the rabbits to examine the serum CRE and BUN concentrations to evaluate the treatment efficacy. The rabbit was fixed, and the back of the rabbit’s ears was sterilized with alcohol cotton. Venipuncture was made using a 24G winged needle (Meditop top scalp vein set, Netherlands) rinsed with heparin with the needle in parallel to the ear artery. The venipuncture site was tapped gently to facilitate blood outflow; 2 mL of blood was collected into a 15-mL centrifuge tube (containing 10% heparin). After centrifuging the blood at 3,000 rpm (1,066 × g) for 10 min, the serum was collected and stored at −80°C. The collected serum was analyzed for CRE and BUN levels with an automatic biochemical analyzer (Vitros 350 Chemistry Analyzer, Johnson & Johnson, CA, United States).

### 2.9 Pathological observations of kidney tissues

All animals were sacrificed 3 weeks after the last injection, i.e., 7 weeks after treatment. A longitudinal section was made to divide the kidney in half and the kidney samples were fixed in 10% neutral formalin, followed by handling with a tissue processor. The fixed tissue was dehydrated and embedded in paraffin, cut into slices of approximately 4 μm, and subjected to hematoxylin and eosin staining. Internal morphological changes in the kidney were observed under an optical microscope.

### 2.10 Statistical methods

All statistical analyses were performed using SPSS Statistics software, version 21.0 (IBM, Armonk, NY, United States). Data were expressed as the mean ± standard deviation (SD). For comparisons of serum BUN levels across groups at each treatment week, one-way analysis of variance (ANOVA) was conducted, followed by *post hoc* Tukey’s multiple comparisons test to determine pairwise differences among groups. The normality of the data distribution was verified using the Shapiro-Wilk test, and homogeneity of variance was assessed using Levene’s test. A p-value of <0.05 was considered statistically significant, and a p-value of <0.01 was considered highly significant. For intergroup comparisons at specific time points, Student’s t-tests were used where applicable, and adjustments for multiple comparisons were applied to control for Type I errors. Statistical results are reported as exact p-values for clarity.

## 3 Results

### 3.1 ADSC characteristics

**FIGURE 1:**
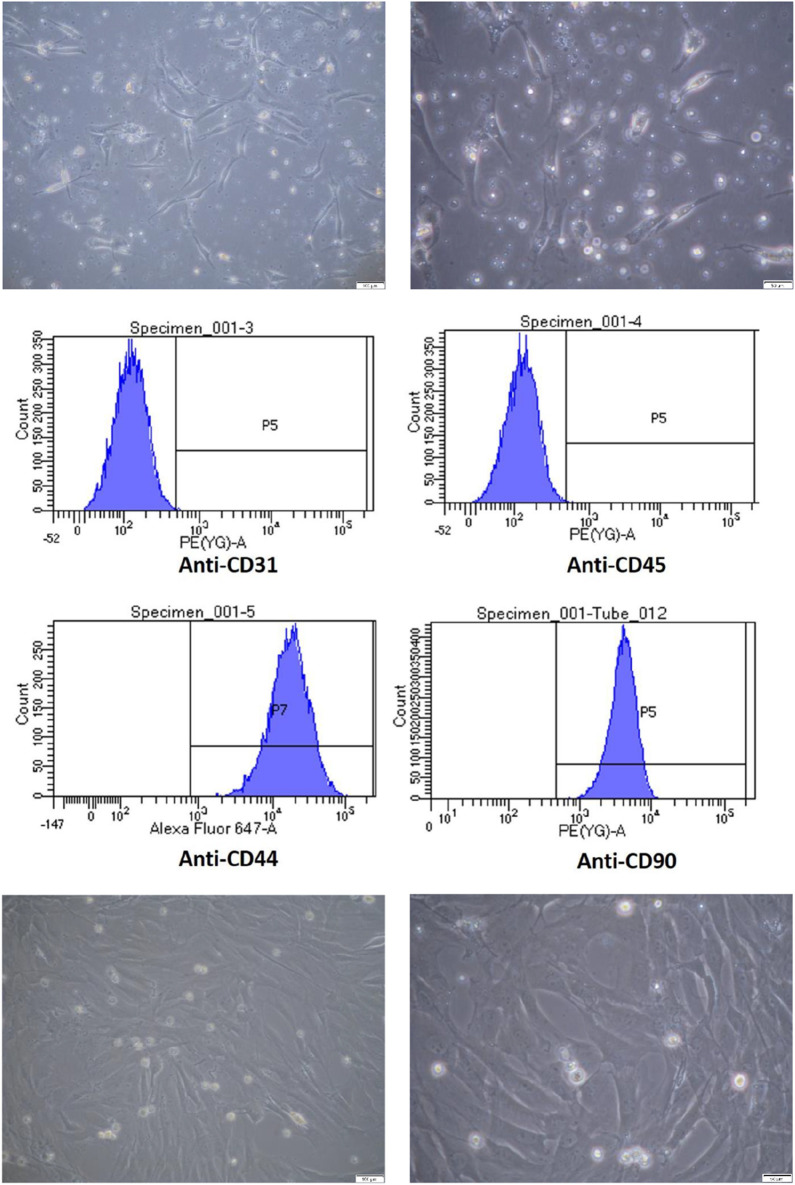
Characterization and Culture of Adipose-Derived Stem Cells (ADSCs). 1-1 Primary culture of ADSCs. ADSCs were cultured and observed under light microscopy at magnifications of 100× (left) and 200× (right), with scale bars indicating 100 µm and 50 μm, respectively. 1-2 Flow cytometric analysis of ADSC surface markers. Flow cytometry analysis confirmed that ADSCs expressed CD44^+^ and CD90^+^, while being negative for CD31 and CD45. 1-3 Subculture of ADSCs up to passage 5. ADSCs were passaged up to the fifth generation and observed under light microscopy at magnifications of 100× (left) and 200× (right), with scale bars indicating 100 µm and 50 μm, respectively.

**FIGURE 2:**
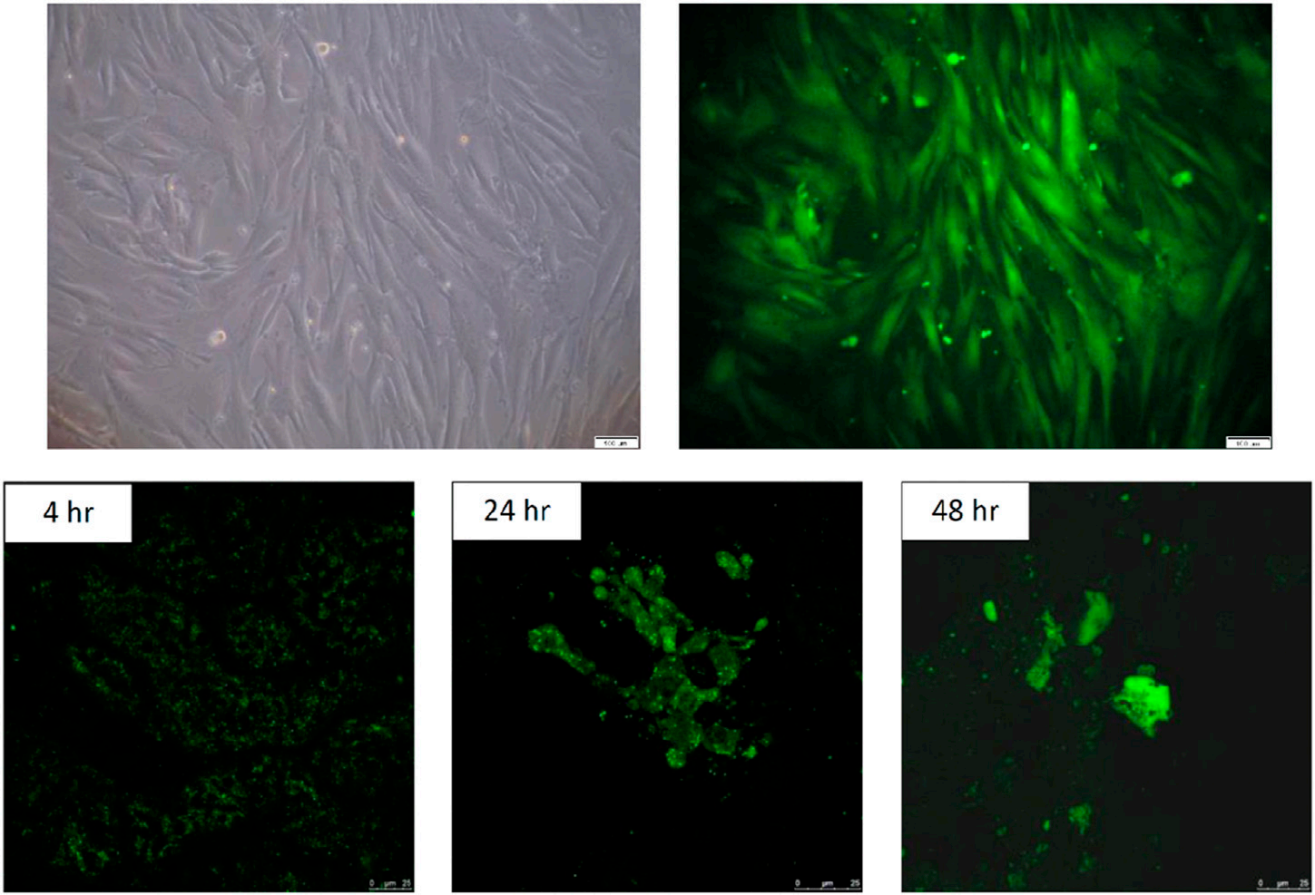
*In vitro* culture and *in vivo* fluorescence imaging of CMFDA-labeled ADSCs. 2-1. *In vitro* culture of CMFDA-labeled ADSCs. Adipose-derived stem cells (ADSCs) were cultured for five passages and labeled with CMFDA. The cells were visualized under light microscopy at 100× magnification. Scale bar = 100 µm. 2-2. *In vivo* fluorescence imaging of CMFDA-labeled fifth-passage ADSCs injected via the BL23 acupoint. Fifth-passage CMFDA-labeled ADSCs were injected into New Zealand white rabbits (n = 3) through the BL23 acupoint. Fluorescence imaging was performed using a laser scanning confocal microscope (TCS SP5 II) to detect labeled cells within renal tissues. Rabbits were sacrificed at 4, 24 and 48 h post-injection, and frozen sections of the left kidney were collected. The intensity of green fluorescence in renal cells increased progressively with time post-injection, indicating a positive correlation between fluorescence intensity and the elapsed time. Scale bar = 25 µm.

**FIGURE 3:**
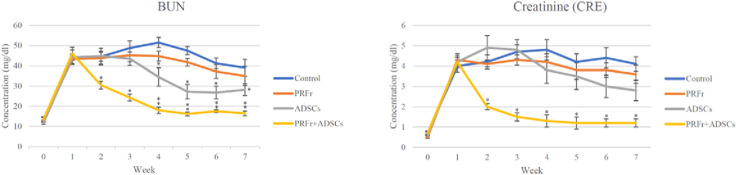
Effects of treatments on BUN and CRE levels in New Zealand white rabbits. 3-1. Changes in BUN levels after 4 injections and 7 weeks of treatment. New Zealand white rabbits were randomized into four groups (n = 6). The control group received PBS injections, while the treatment groups received acupoint injections of PRFr, ADSCs, or PRFr + ADSCs. BUN levels were measured, and statistically significant differences compared to the control group are denoted as follows: *P < 0.05; **P < 0.001. 3-2. Changes in CRE levels after 4 injections and 7 weeks of treatment. New Zealand white rabbits were randomized into four groups (n = 6). The control group received PBS injections, while the treatment groups received acupoint injections of PRFr, ADSCs, or PRFr + ADSCs. CRE levels were measured, with statistical significance compared to the control group indicated by *P < 0.05.

After primary culture and proliferation, the ADSCs showed a spindle shape, as shown in Figure 1-1. In flow cytometry analysis, the phenotype of P2 ADSCs cells identified with CD44 + CD90 + CD34^−^ CD45^−^surface markers are shown in Figure 1-2. The ADSC subcultures after purification are shown in Figure 1-3.

### 3.2 Tracking of fluorescence-labeled ADSCs after acupoint injections

ADSCs stained with CellTracker™ Green CMFDA were spindle-shaped, as shown in Figure 2-1. Experimental white rabbits (n = 3) were injected with fluorescent-labeled CMFDA ADSCs (1 × 10^6^ cells) at the Shenshu acupoint and sacrificed 4, 24, and 48 h later. A laser scanning confocal microscope (TCS SP5 II confocal microscope, Leica, Wetzlar, Germany) was used to capture fluorescent images of the left kidney tissue sections. Green CMFDA was used to mark and locate ADSCs. Figure 2-2 shows the kidney slices of the three groups of experimental rabbits injected with CMFDA-ADSCs. The injection time was positively correlated with green fluorescence in the cells. A high green fluorescence level at 48 h indicated that the CMFDA-ADSC injection at the Shenshu acupoint reached the kidney tissues, particularly located in the renal medulla.

### 3.3 Acupoint injection of ADSCs ± PRFr improved renal functions

The quality of renal filtration can be evaluated by measuring the BUN and CRE levels, which are indices of kidney functions. The nitrogen contained in urea in the blood is known as urea nitrogen and is the residue of protein that has been used as an energy source *in vivo* and is physiologically equivalent to urea. Urea nitrogen is excreted into the urine after being filtered by the glomerulus of the kidney. If the function of renal excretion worsens, the concentration of urea nitrogen in the blood increases; thus, BUN is an important indicator of renal functions. Creatinine is the metabolic product of creatinine in the muscle and is released in the blood. During the initial stage of kidney injury, despite the impaired filtration capacities of the glomeruli, the renal tubules can actively secrete and discharge creatinine. Therefore, blood creatinine does not rise immediately in the early stage of nephropathy, andrenal function has already decreased by more than half when creatinine exceeds the normal range. Therefore, creatinine values alone cannot reflect early renal function changes. The reference value of BUN in rabbit is 17.0–23.5 mg/dL and that of CRE is 0.8–1.8 mg/dL (Harkness and Wagner, 1983). Values were used for reference only and did not represent the normal value in any groups). In this study, changes in blood urea nitrogen (BUN) and serum creatinine (CRE) levels, both markers of renal function, are shown in Figures 3-1, 3-2, Tables 1, 2. At baseline, no significant differences were observed between groups. By Week 2, the PRFr + ADSCs group showed a significant reduction in BUN (30.5 ± 3.8 mg/dL, P < 0.05) and CRE (2.0 ± 0.3 mg/dL, P < 0.05) levels compared to the control. This trend continued, with the PRFr + ADSCs group exhibiting the most pronounced decreases by Week 4 (BUN: 18.0 ± 3.1 mg/dL, P < 0.001; CRE: 1.3 ± 0.6 mg/dL, P < 0.05). The ADSCs group also showed a significant reduction in BUN at Week 4 (34.5 ± 9.1 mg/dL, P < 0.05). By Week 7, the PRFr + ADSCs group maintained significantly lower BUN (16.5 ± 2.3 mg/dL, P < 0.001) and CRE (1.2 ± 0.4 mg/dL, P < 0.05) levels, indicating a superior improvement in renal function compared to other groups. These results suggest that the combination of PRFr and ADSCs significantly enhances renal function, as reflected by the consistent reductions in both BUN and CRE levels.

**TABLE 1:** Changes in blood urea nitrogen (BUN) levels of New Zealand white rabbits after 4 injections and 7 weeks of treatment. New Zealand white rabbits were randomized into 4 groups (n = 6). No treatment was administered to the control group, whereas acupoint injections of PRFr, ADSCs, and PRFr + ADSCs were performed in the treatment groups.

Week of treatment	Mean BUN mg/dL(n = 6)			
	Control	PRFr	ADSCs	PRFr + ADSCs
0	13.2 ± 2.4	12.8 ± 0.8	11.7 ± 1.5	13.5 ± 2.7
1	43.3 ± 5.0	43.3 ± 4.5	44.2 ± 7.2	46.0 ± 6.4
2	44.5 ± 4.5	43.8 ± 6.8	44.8 ± 7.9	30.5 ± 3.8*
3	48.8 ± 7.1	45.2 ± 6.5	43.5 ± 6.6	24.3 ± 3.5*
4	51.5 ± 5.1	44.8 ± 4.9	34.5 ± 9.1*	18.0 ± 3.1**
5	47.5 ± 4.0	41.8 ± 3.8	27.2 ± 7.1*	16.2 ± 2.1**
6	41.2 ± 5.2	37.2 ± 6.8	26.8 ± 6.3*	17.5 ± 1.4**
7	39.0 ± 8.5	35.0 ± 9.7	28.2 ± 5.8*	16.5 ± 2.3**

*Indicates a statistically significant difference compared to the control group (P < 0.05); **Indicates a highly significant difference compared to the control group (P < 0.001).

**TABLE 2:** Changes in serum creatinine (CRE) levels of New Zealand white rabbits after 4 injections and 7 weeks of treatment. New Zealand white rabbits were randomized into 4 groups (n = 6). No treatment was administered to the control group, whereas acupoint injections of PRFr, ADSCs, and PRFr + ADSCs were performed in the treatment groups.

Week of treatment	Mean CRE level mg/dL(n = 6)			
	Control	PRFr	ADSCs	PRFr + ADSCs
0	0.7 ± 0.2	0.5 ± 0.1	0.6 ± 0.1	0.7 ± 0.1
1	4.0 ± 0.6	4.3 ± 0.6	4.2 ± 0.6	4.2 ± 0.4
2	4.2 ± 0.7	4.1 ± 0.3	4.9 ± 1.2	2.0 ± 0.3*
3	4.7 ± 0.7	4.3 ± 0.5	4.8 ± 1.0	1.5 ± 0.4*
4	4.8 ± 1.0	4.2 ± 0.8	3.8 ± 1.3	1.3 ± 0.6*
5	4.2 ± 0.8	3.8 ± 0.9	3.5 ± 1.3	1.2 ± 0.6*
6	4.4 ± 1.0	3.8 ± 0.7	3.0 ± 1.1	1.2 ± 0.4*
7	4.1 ± 0.7	3.6 ± 0.9	2.8 ± 1.0	1.2 ± 0.4*

*Indicates a statistically significant difference compared to the control group (P < 0.05).

### 3.4 Histological analysis

**FIGURE 4:**
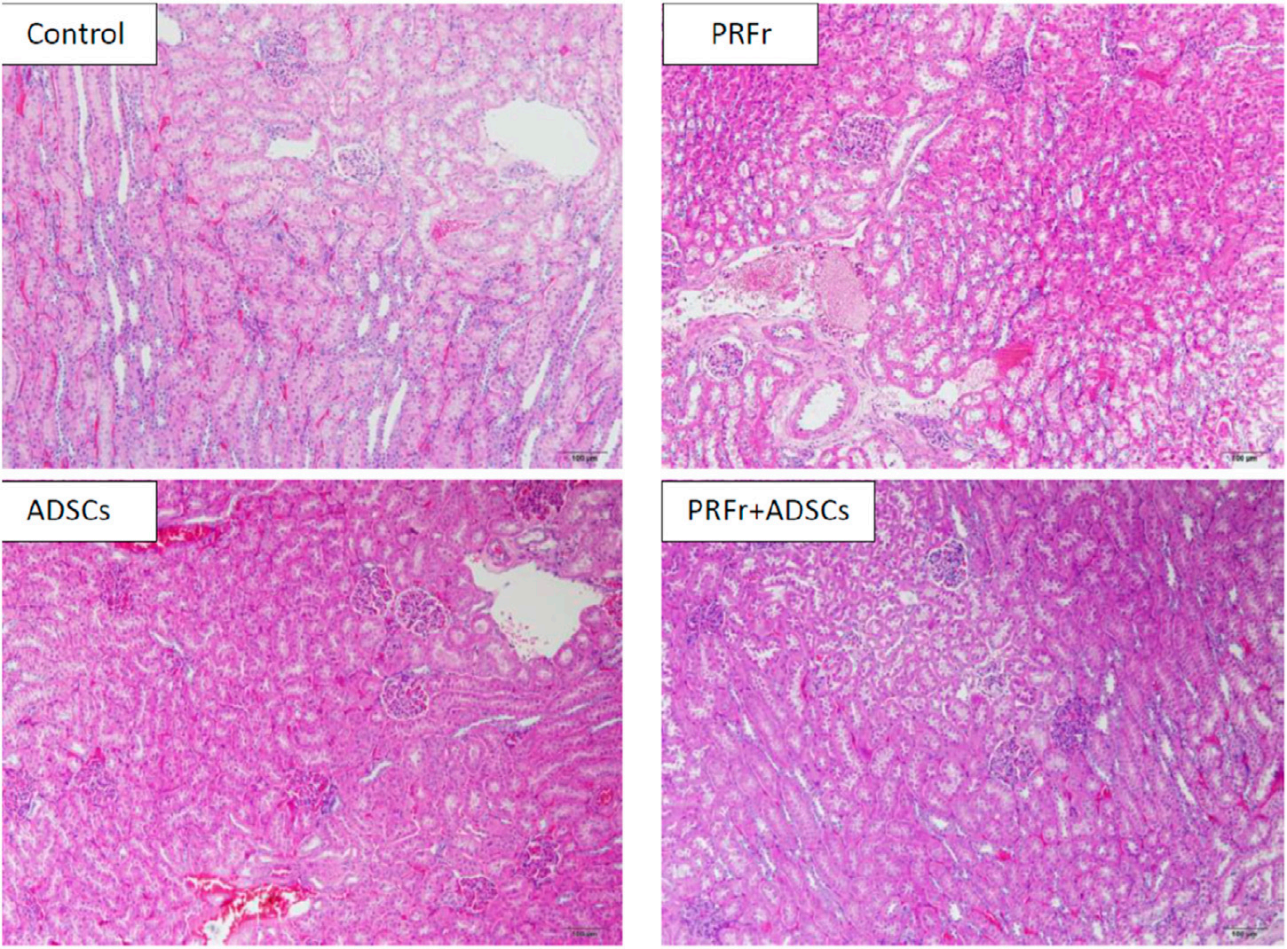
The structural integrity and inflammatory status of the kidney were assessed using Hematoxylin and Eosin staining. The images reveal that in the PRFr + ADSCs treatment group, the glomerular morphology remained intact, and the renal tubular epithelial cells exhibited no signs of edema or vacuolar changes. These results are superior compared to the groups treated with either ADSCs or PRFs alone. Magnified at 100×, scale bar = 100 μm.

Figure 4 shows the histological images of the sections from the four kidney injury groups after repair. Hematoxylin and eosin staining showed that in the PRFr + ADSC treatment group, the glomeruli were intact with no edema or vacuole-like changes in the epithelial cells of renal tubules and no obvious inflammatory cells in the surrounding area. No tubular obstruction was observed in the renal tubules. In the ADSC treatment group, the glomeruli structure was intact with tubular obstruction observed in few renal tubules, along with local edema and thickening in the epithelium of a few renal tubules. In the PRFr treatment group, part of the glomeruli shrank, with local edema and thickening observed in the renal tubular epithelium. Vacuole-like changes as well as degeneration and necrosis of some epithelial cells were observed. In the control group, the glomeruli shrank, and inflammatory cell effusion was observed. In the renal tubules, edema of the epithelium and necrosis with vacuole-like changes were observed with tubular obstruction and disintegration of part of the renal tubulars.

## 4 Discussion

CKD is a risk factor for cardiovascular diseases. Rapid developments in stem cell-related research have been made in recent years, including in the culture of pluripotent stem cells *in vitro* with the goal of differentiating these cells into various tissues and organs to repair damage in the human body. Mesenchymal stem cells (MSCs) are an important group of stromal cells that can self-renew and are multipotent *in vitro*. Bone marrow-derived MSCs (BMSCs) are characterized by invasive acquisition with side effects such as pain, infection, and bleeding at the puncture site. BMSCs can be cultured and proliferated *in vitro*, but their growth is slow and they show low yield (Dominici et al., 2009). ADSCs are an attractive source of autologous cells for various therapeutic purposes and can be collected from subcutaneous adipose tissue using conventional liposuction. They are easy to separate, relatively abundant, uncontroversial, and can be cultured and proliferated *in vitro* (Zuk et al., 2001). ADSCs have similar cell phenotypes as BMSCs but show higher yields and proliferation rates compared to BMSCs (Liao et al., 2010; Nery et al., 2013). ADSCs secrete a variety of cytokines and growth factors with paracrine effects for mediating and stimulating tissue repair and regeneration (Murphy et al., 2013). However, various challenges remain to using these cells. Even if stem cells can be cultured *in vitro*, no method is available for producing fully functional tissues when inducing the differentiation of these stem cells. Some tissues require a three-dimensional structure and some require secretory functions. If stem cells can be induced to differentiate into tissues with the correct structure and functions, they can be transplanted into the body to treat diseases.

Furthermore, the platelet concentrate PRP/PRF for local infiltration has been used as an adjuvant or regenerative medicine preparation in most medical fields, particularly in plastic surgery, cardiovascular surgery, surgical reconstruction, sports medicine, dentistry, and oral implantology. Growth factors, healing proteins, cell signals, and immune cells are present in the human blood which are part of the natural healing process and can be concentrated and applied or injected in wounds or at surgical sites to promote healing. PRP contains key growth factors and mediators that promote the proliferation, migration, and differentiation of stem cells during tissue regeneration and have been confirmed as a biomaterial useful for tissue regeneration (Liao et al., 2015; Ramos-Torrecillas et al., 2014). Many studies have shown that transplantation combining ADSCs and PRP has multiple effects and promotes tissue regeneration (Pak et al., 2013; Tajima et al., 2015). PRP contains various cytokines, including platelet-derived growth factor, vascular endothelial growth factor, transforming growth factor-beta, and insulin-like growth factor 1 (Boswell et al., 2012). Some studies have reported that PRP can enhance colony formation, maintain the adipogenic, chondrogenic, and osteogenic differentiation potential of stem cells, and sustain their immunosuppressive properties (Duan et al., 2011). Furthermore, literature indicates that PRP can attract and enhance the capabilities of MSCs through exosomes, thereby promoting the initial healing of wounds (Guo et al., 2017). In fact, studies have shown that PRP can stimulate the secretion of exosomes from MSCs, leading to therapeutic effects, such as in the treatment of acute kidney injury (Ji et al., 2021). Another platelet concentrate preparation, PRF, aggregates platelets and releases cytokines in the clot because of the polymerization of fibrin gel (Dohan et al., 2006). Therefore, growth factors in the fibrin clot participate in the polymerization of platelets and fibrin and are gradually released over time (Lundquist et al., 2008). The released PRF extracted by squeezing the PRF clot (serum exudate exhibited as clots) is known as PRFr, which is rich in growth factors such as platelet-derived growth factor, transforming growth factor, VEGF, epithelial growth factor, insulin-like growth factor, fibroblast growth factor, and nerve growth factor, white blood cells, lipids, and proteins such as vitronectin and fibronectin. The exudate can be used to hydrate graft materials and promote cell proliferation by inducing cell transformation, increase matrix formation, bone formation, and collagen synthesis, stimulate granulation and angiogenesis, as well as accelerate wound healing and tissue regeneration by inducing cell differentiation to eliminate necrotic tissues. PRFr is separated from the PRF gel using static and centrifugal processes. Many *in vitro* tests have demonstrated that PRF shows higher growth factor release compared to PRP (He et al., 2009). Therefore, PRFr appears to be more promising for stimulating cell-related activities.

In 2010, Li et al. obtained hematopoietic stem cells from injured kidneys that produced high concentrations of pro-angiogenic cytokines, including VEGF-A (Li et al., 2010). In 2014, Xing et al. cultured MSCs and transplanted them into a mouse model of IR injury. MSC infusion can promote kidney repair in various manners, including by by exerting anti-inflammatory and anti-apoptosis effects (Xing et al., 2014). In addition, MSCs secrete high concentrations of pro-angiogenic and growth factors, including hepatocyte growth factor, VEGF-A, and insulin-like growth factor-1. MSCs participate in kidney repair through paracrine signaling (Xing et al., 2014). We previously demonstrated in several studies that transplantation combining ADSCs and PRFr significantly enhanced bone formation in osteoporotic mice (Chuang et al., 2020) and promoted regeneration of the injured sciatic nerve in rats (Sheu et al., 2020).

Acupuncture, as a treatment for CKD, promotes the recovery of kidney disease by promoting the flow of Qi to regulate blood and kidney functions. Shenshu is the Shu acupoint on the back side of the bladder meridian. Acupuncture at this point can smooth the Qi of the kidney and treat kidney-related diseases. Zhang et al. suggested that electroacupuncture at Shenshu inhibits the Wnt/β-catenin signaling pathway by decreasing β-catenin expression in the kidney tissue, thereby preventing the progression of CKD and restoring renal function (Zhang et al., 2014). Acupoint injection therapy can retain the drug at the Shu acupoint for a longer time, which may enhance the therapeutic effect on the meridian and Shu acupoint through prolonged stimulation. In addition, injection of drugs at the Shu acupoint activated the beneficial regulatory effect of Shu on the viscera and improved the development of the body’s pathological state, reducing the required drug dosage and improving efficacy (Huang et al., 2019). Shenshu is the acupoint of the bladder meridian of foot-Taiyang and where the kidney’s Qi is injectedfornourishing the kidney and strengthening the waist, promoting Yang and diuresis, andimprovinghearing and vision. Acupuncture at this site can invigorate the kidney and remove dampness, strengthen the waist and spine, and replenish water and promote fireto treat kidney diseases, bladder diseases, and collateral diseases. We are trying to combine the therapeutic properties of ADSCs and PRFr and use acupuncture for transplantation to see if it is beneficial for the treatment of acute kidney injury. This approach was based on the self-proliferation capacity and potential of ADSCs to differentiate into several cell types, combining with PRFr as a carrier of stem cells because of its high levels of growth factors and cytokines. We developed a treatment method based on TCM involving acupoints and meridians for stem cell treatment of a kidney injury model to overcame the limitations and surgical risks of pluripotent stem cell transplantation.Compared with PRP, this method allowed for the release of more growth factors to stimulate cell-related activities.

PRFr and ADSCs were injected at the Shenshu acupoint (BL23) to treat the injured kidney. This approach enhanced the repair of renal tubules, thereby improving renal functions. Paracrine and endocrine effects may have helped to protect the kidneys. Gharaibeh et al. also proposed that terminal differentiation of implanted stem cells was not the major determinant of cell regeneration potential, whereas the paracrine effect conferred by transplanted cells played a greater role during regeneration (Gharaibeh et al., 2011). Xing et al. also pointed out that the role of MSCs may be related to the pluripotency of stem cells, including increased secretion of paracrine factors as well as improved angiogenesis, anti-inflammatory activity, and anti-apoptotic effects (Xing et al., 2014). A sharp increase in serum CRE and BUN levels was observed at 1 week after establishing the kidney injury model. The CRE and BUN levels of the PRFr + ADSCs treatment group decreased significantly during the second week after transplantation. After 7 weeks of treatment, PRFr + ADSCs showed the best kidney repair outcomes, followed by ADSCs alone (*p* < 0.01) and PRFr alone (*p* < 0.05). In the PRFr + ADSCs treatment group, the morphology of the glomeruli was intact, with no edema or vacuole-like changes in the epithelial cells of the renal tubules and no obvious inflammatory cells in the surrounding area. No tubular obstruction was observed in the lumen of the renal tubules. In rabbits with IR after AKI, stem cell acupoint therapy significantly repaired damaged cells, with PRFr + ADSCs showing the best outcome.Based on our results, injection of PRFr, ADSCs, and PRFr + ADSCs at Shenshu acupoint (BL23) can be used to repair damaged renal cells and decrease serum CRE and BUN levels as a potential treatment for kidney injury.

## Data Availability

The original contributions presented in the study are included in the article/Supplementary Material, further inquiries can be directed to the corresponding authors.
