# Supplementary material for: Effect of injecting adipose stem cells combined with platelet-rich fibrin releasate at Shenshu acupoint (BL23) on acute kidney injury in rabbits
Source: Front Pharmacol. 2025 Mar 12;16:1409056. doi: 10.3389/fphar.2025.1409056 (PMC11936987; doi:10.3389/fphar.2025.1409056)
Supplement: Supplementary file 1 [file DataSheet1.docx]

Supplement 1 Acupuncture injection at Shenshu BL23 with fluorescence-labeled

ADSCs

Supplement 2 Surgery establishing the rabbit model of ischemia reperfusion after acute kidney injury. A hemostatic forceps with a thin plastic catheter at the front was used to clamp the renal artery for 45 min, and then the clamp was released (n = 2)
